# Neuroprotective Effects of the Multitarget Agent AVCRI104P3 in Brain of Middle-Aged Mice

**DOI:** 10.3390/ijms19092615

**Published:** 2018-09-04

**Authors:** Julia Relat, Julio Come, Belen Perez, Pelayo Camps, Diego Muñoz-Torrero, Albert Badia, Lydia Gimenez-Llort, M. Victòria Clos

**Affiliations:** 1 Neuroscience Institute, Autonomous University of Barcelona, 08193 Barcelona, Spain; Julirelat@hotmail.com (J.R.); jcome84@gmail.com (J.C.); Belen.perez@uab.es (B.P.); albert.badia@uab.cat (A.B.); Lidia.Gimenez@uab.cat (L.G.-L.); 2 Department of Pharmacology, Therapeutic and Toxicology, Autonomous University of Barcelona, 08193 Barcelona, Spain; 3 Laboratory of Pharmaceutical Chemistry (CSIC Associated Unit), Faculty of Pharmacy and Food Sciences, and Institute of Biomedicine (IBUB), University of Barcelona, 08028 Barcelona, Spain; camps@ub.edu (P.C.); dmunoztorrero@ub.edu (D.M.-T.); 4 Department of Psychiatry and Forensic Medicine, Autonomous University of Barcelona, 08193 Barcelona, Spain

**Keywords:** acetylcholinesterase inhibitors, huprine derivatives, AVCRI104P3, apoptosis, neuroprotection, neuroinflammation

## Abstract

Molecular factors involved in neuroprotection are key in the design of novel multitarget drugs in aging and neurodegeneration. AVCRI104P3 is a huprine derivative that exhibits potent inhibitory effects on human AChE, BuChE, and BACE-1 activities, as well as on AChE-induced and self-induced Aβ aggregation. More recently, cognitive protection and anxiolytic-like effects have also been reported in mice treated with this compound. Now, we have assessed the ability of AVCRI104P3 (0.43 mg/kg, 21 days) to modulate the levels of some proteins involved in the anti-apoptotic/apoptotic processes (pAkt1, Bcl2, pGSK3β, p25/p35), inflammation (GFAP and Iba1) and neurogenesis in C57BL/6 mice. The effects of AVCRI104P3 on AChE-R/AChE-S isoforms have been also determined. We have observed that chronic treatment of C57BL/6 male mice with AVCRI104P3 results in neuroprotective effects, increasing significantly the levels of pAkt1 and pGSK3β in the hippocampus and Bcl2 in both hippocampus and cortex, but slightly decreasing synaptophysin levels. Astrogliosis and neurogenic markers GFAP and DCX remained unchanged after AVCRI104P3 treatment, whereas microgliosis was found to be significantly decreased pointing out the involvement of this compound in inflammatory processes. These results suggest that the neuroprotective mechanisms that are behind the cognitive and anxiolytic effects of AVCRI104P3 could be partly related to the potentiation of some anti-apoptotic and anti-inflammatory proteins and support the potential of AVCRI104P3 for the treatment of brain dysfunction associated with aging and/or dementia.

## 1. Introduction

The constant increase of individual and social aging is leading to a “more years, better lives” paradigm with particular potentials and challenges. However, this demographic change also leads us to confront the concurrent increase of prevalence and impact of many age-related neurologic and neuropsychiatric disorders, including late-onset neurodegenerative diseases such as Alzheimer’s and Parkinson’s diseases.

In this context, the central cholinergic system plays a key role as the most important circuit involved in cognitive function and because the integrity of cholinergic system in older adults underlies inter-individual variability in their memory function [1]. Moreover, a recent systematic review on risk factors and prevention of cognitive decline in later life reveals that individuals with cognitive decline are at a higher risk of progressing to mild cognitive impairment and dementia [2]. It is also known that molecular aging of the brain overlaps with biological pathways implicated in multiple brain disorders. Thus, different strategies have been considered to improve the cognitive process in aging and also to treat dementia. So far, the best pharmacological tools to attenuate cognitive impairment in patients with mild-to-moderate Alzheimer’s disease (AD) are acetylcholinesterase inhibitors (AChEIs) [3,4,5]. While these drugs are currently used as a symptomatic treatment, to improve or at least maintain central cholinergic function [6], recent lines of evidence suggest that cholinesterase inhibitors may have broader functions beyond enzyme inhibition [7]. New data suggest the involvement of AChEIs in modulating glial activation [8], cerebral blood flow, inflammation, amyloid cascade, and tau phosphorylation. It has therefore been speculated that some actions of AChEIs could affect the underlying disease processes in AD, and, hence, that pharmacological manipulation with AChEIs might affect long-term disease progression [9].

In line with this evidence, we have demonstrated behavioral and disease-modifying effects of huprine X in animal models of normal aging and AD-related cognitive impairment. In middle-aged mice the anticholinesterasic huprine X [10] facilitates learning and memory in the Morris water maze test and improves some indicators of emotionality without inducing adverse effects, anxiety-like behaviors or affecting motor activity [11]. Activation of downstream PKC/MAPK signalling pathways may underlie these behavioral effects, as well as the stimulation of the non-amyloidogenic processing of APP [11]. Huprine X also improved cognition in six-month-old 3xTg-AD [12], and biochemical data, such as an increase in synaptophysin and a decrease in Aβ1-40, providing further evidence that this drug can modulate some of the fundamental processes that contribute to neurodegeneration [13]. Recently, we have also shown that HX treatment ameliorates the toxic effects in the kainic acid mouse model [14]. We have also demonstrated disease-modifying effects in several families of huprine-based hybrid compounds, which, apart from displaying very potent AChE inhibitory activity (IC_50_ values towards human AChE in the single-digit nanomolar range), modulate several key factors involved in the underlying mechanisms of AD when administered to APP/PS1 mice, including amyloid burden, neuroinflammation and epileptogenic activity, and enhance cognition [15,16,17]. Despite the outstanding in vivo pharmacological profile of these and other multitarget hybrid compounds [18], the launching of a disease-modifying anti-Alzheimer drug remains elusive so far. In this scenario, a continuous intensive search for novel drug candidates with potential to positively modify the natural course of AD is a dire need.

In this context, in the frame of our recent efforts in AD drug design a family of hybrid compounds that featured pharmacophoric moieties of huprine and the anti-Alzheimer drug donepezil was developed as a novel class of multitarget agents [19,20]. Within this novel structural class, compound AVCRI104P3 deserves special attention. This lead compound exhibits an interesting in vitro multitarget biological profile. It shows a potent in vitro inhibitory activity against human AChE (IC_50_ = 5.4 nM) and human BChE (IC_50_ = 88 nM) and a moderately potent inhibitory activity of BACE-1 (29% inhibition at 5 µM), and Aβ aggregation (28% inhibition at 10 µM) [19]. In an in vivo study, we recently found that AVCRI104P3 exerted pro-cognitive effects in middle-aged mice, improving both short- and long-term processes and leading to a fast and efficient acquisition of the place task in the Morris water maze [21]. In addition to the cognitive enhancement, AVCRI104P3 exerted anxiolytic-like effects with a lack of adverse effects. Cognitive enhancement through the stimulation of central cholinergic neurotransmission has been regarded to counteract the central effects of the aging process and it is also one of the main strategies for AD treatment. Taking into account previous results and the fact that oxidative stress, apoptosis, neurotoxicity, and inflammation are involved in the aging process, in the present study we have characterized the ex vivo effects of AVCRI104P3 on the levels of several proteins related to these processes. Specifically, we have determined the expression of Akt1, Bcl2, GSK3β, synaptophysin, p25/p35 ratio, and caspases involved in apoptotic and survival pathways in both hippocampus and cortex, and GFAP, Iba1, and DCX as glial and neurogenesis markers, respectively. The expression of AChE variants (AChE-R, AChE-S) was also analyzed, since AChE isoforms expression plays a role in neurotoxicity/neuroprotection mechanisms [22,23]. The results of these studies indicate that the multitarget AVCRI104P3 shows significant protective effects that make it a promising therapeutic compound for brain dysfunction underlying natural aging and/or dementia.

## 2. Results

### 2.1. Effects of AVCRI104P3 on Akt1, Bcl2, GSK3β, Caspases, p25/p35 and Synaptophysin Expression

**Figure 1:**
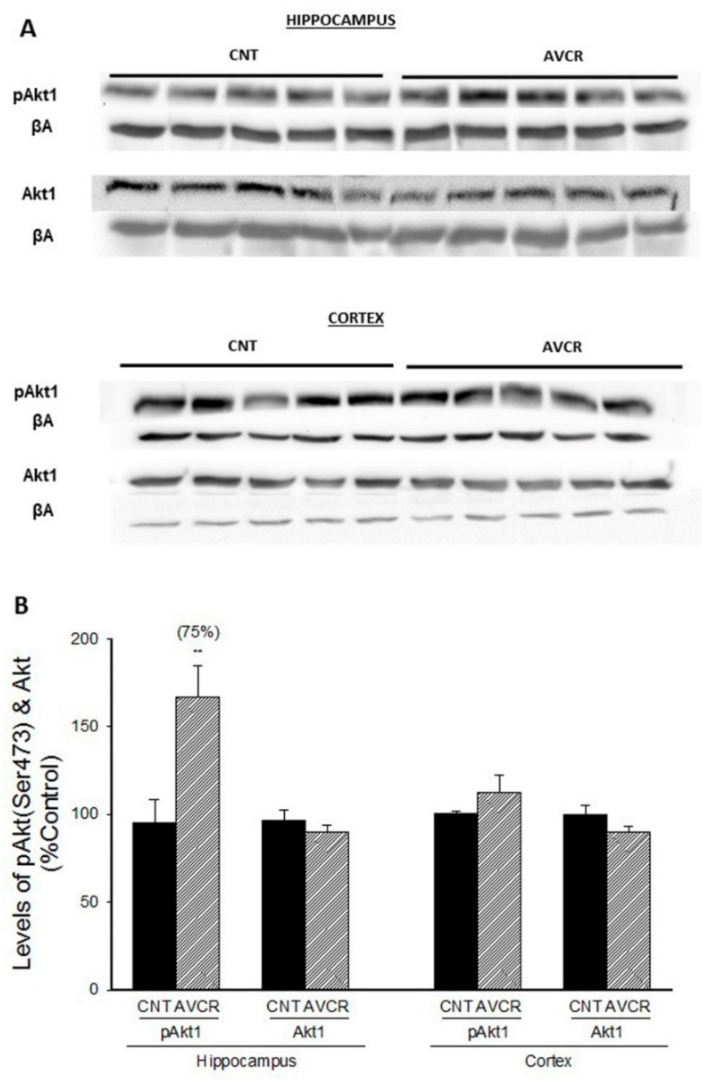
Effects of AVCRI104P3 on pAKt (Ser473) and Akt in the hippocampus and cortex of 12-month-old 129/Sv × C57BL/6 male mice. (**A**) Representative Western blot images of pAKt (Ser473) and Akt β-actin were used as internal control. (**B**) Photodensitometric quantification of WB experiments was used to evaluate changes in pAkt (Ser473) and Akt expression. The results are the mean ± SEM of 3–4 experiments (5 mice/treatment group). The statistical analysis used was one-way ANOVA followed by Dunnett’s test ** *p* < 0.01 vs. CNT.

It is well known that Akt is involved in the modulation of several factors such as GSK3β, caspases, and BAD [24]. We have determined pAKt1 and Akt1 expression in both hippocampus and cortex and found a significant increase in the expression of activated phosphorylated pAkt1 in hippocampus of mice treated with AVCRI104P3 compared with control mice (Figure 1; 75% increase, *p* < 0.01; *n* = 5).

**Figure 2:**
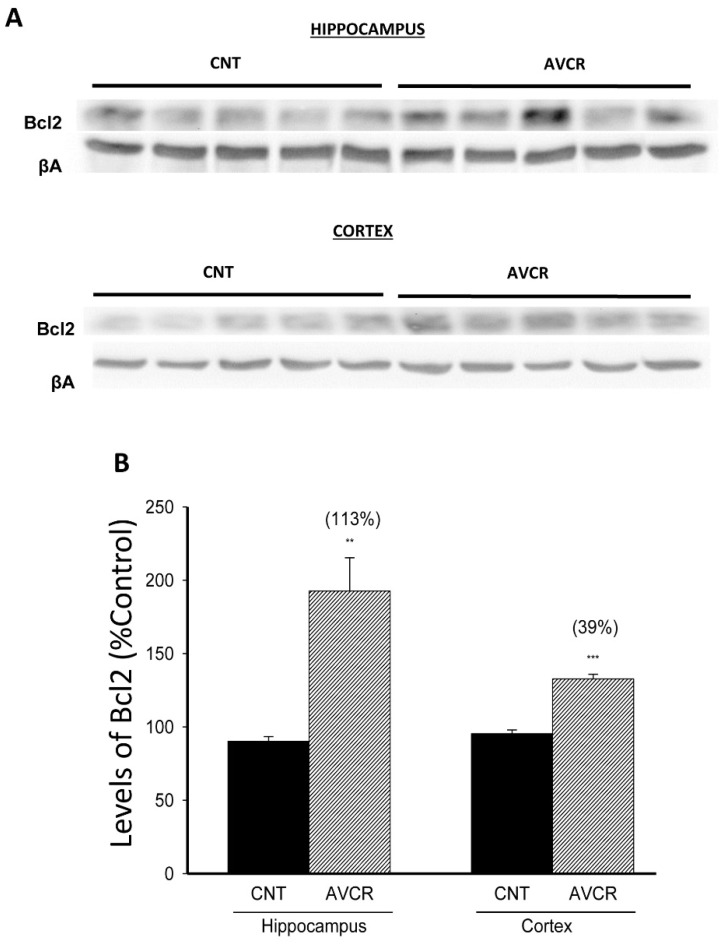
Effect of AVCRI104P3 on Bcl2 in the hippocampus and cortex of 12-month-old 129/Sv × C57BL/6 male mice. (**A**) Representative Western blot images of Bcl2. β-actin was used as internal control. (**B**) Representation of the photodensitometric analysis of the Bcl2 levels. The results are the mean ± SEM of 3–4 experiments (5 mice/treatment group). The statistical analysis used was one-way ANOVA followed by Dunnett’s test, ** *p* < 0.01 vs. CNT, *** *p* < 0.001 vs. CNT.

Bcl2 is a protein family that governs the permeability of mitochondrial membrane and has an important role in both apoptotic and anti-apoptotic processes. When we analysed the effect of AVCRI104P3 on the anti-apoptotic protein Bcl2, we observed a significant increase of protein expression in both hippocampus (113%, *p* < 0.01) and cortex (39%, *p* < 0.01) (Figure 2).

**Figure 3:**
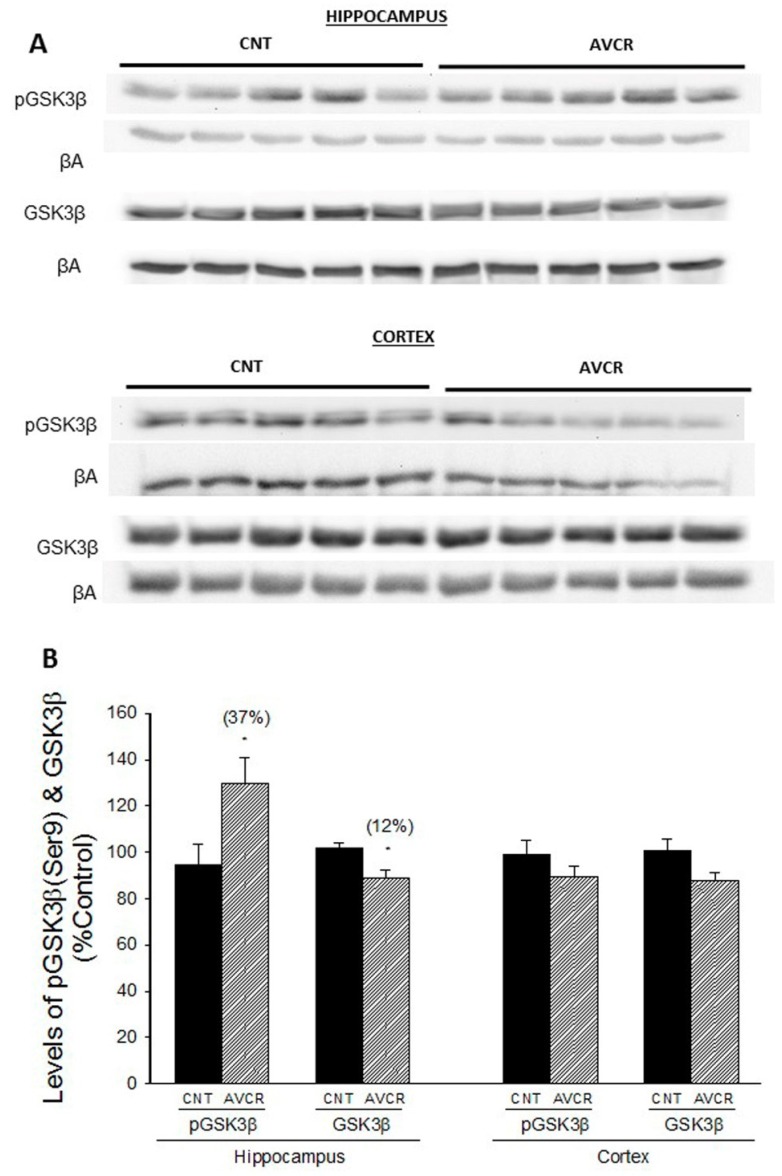
Effect of AVCRI104P3 on pGSK3β (Ser9) and GSK3β in the hippocampus and cortex of 12-month-old 129/Sv × C57BL/6 male mice. (**A**) Representative Western blot images of pGSK3β (Ser9) and GSK3β. β-actin were used as internal control. (**B**) Photodensitometric quantification of WB experiments was used to evaluate changes in pGSK3β and GSK3β expression. The results are the mean ± SEM of 3–4 experiments (5 mice/treatment group). The statistical analysis used was one-way ANOVA followed by Dunnett’s test, * *p* < 0.05 vs. CNT.

Regarding the regulatory kinase of glycogen metabolism (GSK3β), AVCRI104P3 treatment resulted in a significant increase in the phosphorylated form pGSK3β (37%, *p* < 0.05) and a decrease in GSK3β (12%, *p* < 0.05) in hippocampus (Figure 3), without inducing any change in the expression of this protein in cortex.

The effects of AVCRI104P3 treatment on the activity of caspases, a family of proteases that are activated under apoptotic stimuli, were also studied. We have found that the drug did not modify caspase 3/7 activity in cortex (control = 8939 ± 254; AVCRI104P3 = 9231 ± 686; luminescence units).

**Figure 4:**
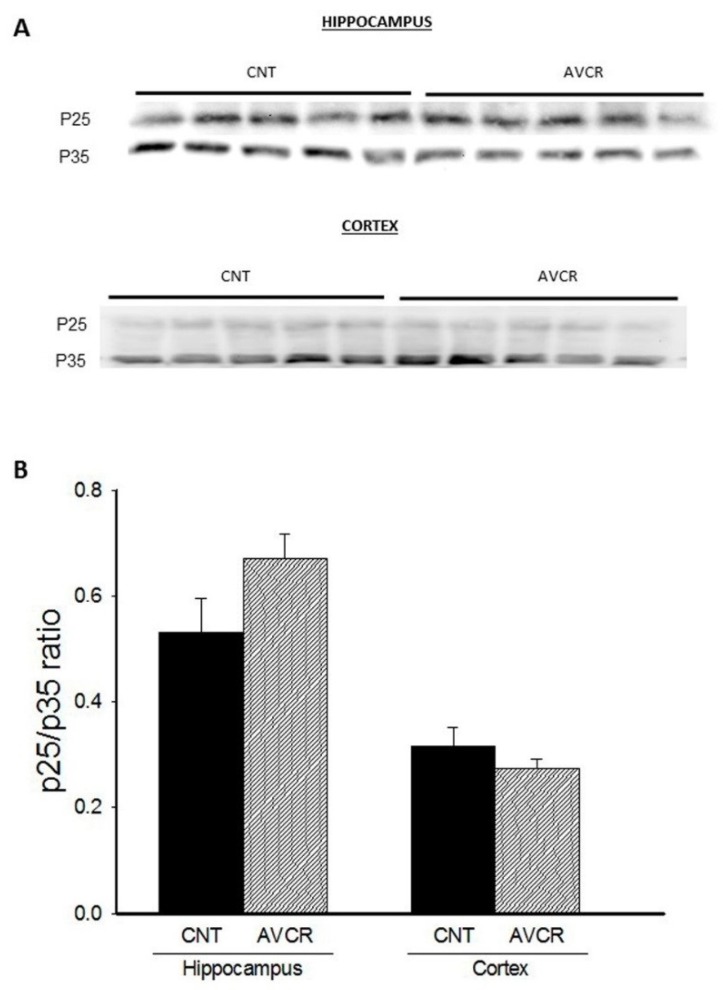
Effect of AVCRI104P3 on p25/p35 ratio in the hippocampus and cortex of 12-month-old 129/Sv × C57BL/6 male mice. (**A**) Representative Western blot images of p25/p35 ratio. β-actin were used as internal control. (**B**) Photodensitometric quantification of WB experiments was used to evaluate changes in the p25/p35 ratio. The results are the mean ± SEM of 3–4 experiments (5 mice/treatment group). The statistical analysis used was one-way ANOVA followed by Dunnett’s test.

The p25/p35 ratio is an indicative parameter of the inactivation/activation of Cyclin-dependent kinase-5 (CDK5), a protein that seems to be involved in the modulation of synaptic plasticity. In this study, we have found that the p25/p35 ratio was not significantly modified upon AVCRI104P3 treatment either in hippocampus or in cortex (Figure 4).

**Figure 5:**
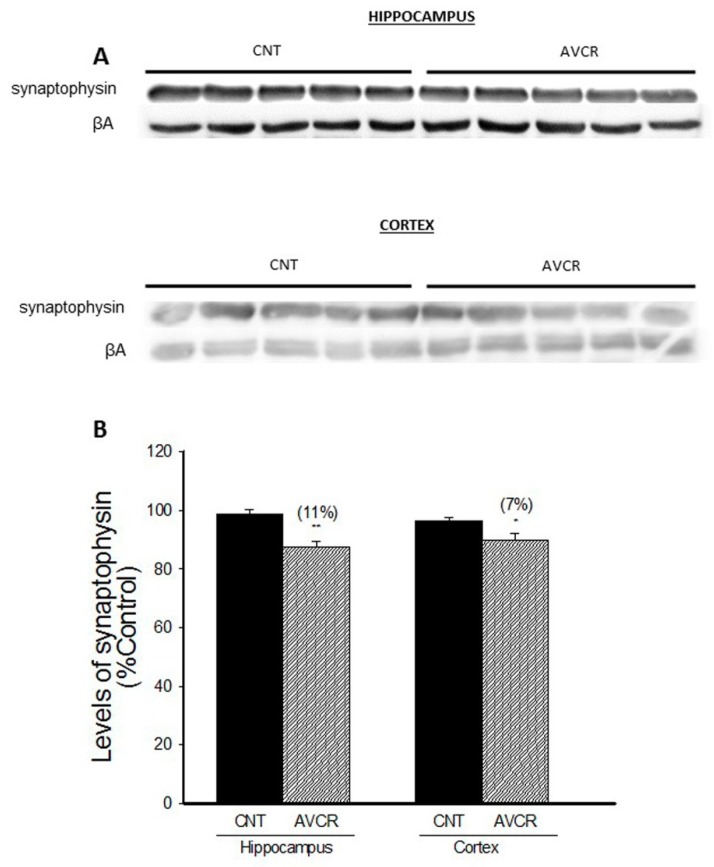
Effect of AVCRI104P3 on synaptophysin in the hippocampus and cortex of 12-month-old 129/Sv × C57BL/6 male mice. (**A**) Representative Western blot images of synaptophysin. β-actin were used as internal control. (**B**) Photodensitometric quantification of WB experiments was used to evaluate changes in synaptophysin expression. The results are the mean ± SEM of 3–4 experiments (5 mice/treatment group). The statistical analysis used was one-way ANOVA followed by Dunnett’s test, * *p* < 0.05, ** *p* < 0.01 vs. CNT.

Synaptophysins are a family of proteins located in presynaptic vesicles. They are considered a marker of synaptogenesis and neuroplasticity. We have found that AVCRI104P3 treatment led to a slight but significant decrease in the levels of this synaptic protein in hippocampus (11%, *p* < 0.01) and in cortex (7%, *p* < 0.05) (Figure 5).

### 2.2. Effects of AVCRI104P3 on Neuroinflammation (GFAP, Iba-1) and Neurogenesis (DCX) in the Brain

**Figure 6:**
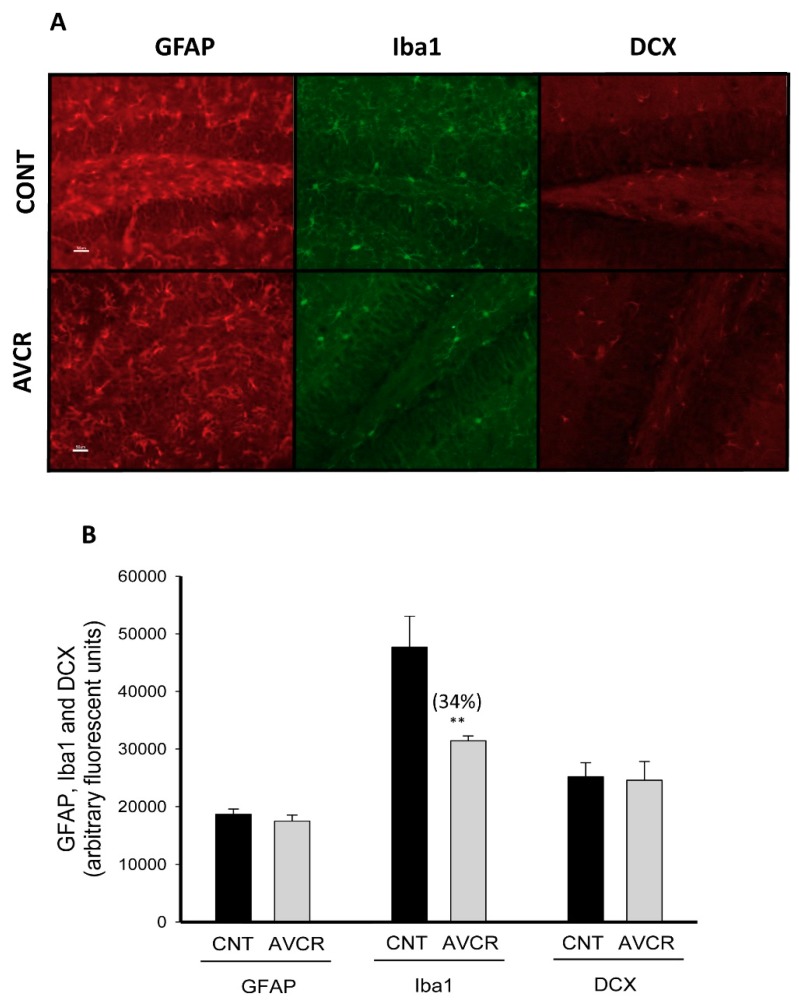
Effect of AVCRI104P3 on GFAP, Iba1 and DCX in the hippocampus (dentate gyrus, DG) of 12-month-old 129/Sv × C57BL/6 male mice. (**A**) Representative images of immunohistochemical studies for GFAP, Iba1 and DXC. Scale bar: 50 µm. (**B**) Semi-quantitative analysis of optical density using the free Image J 1.49 programme. Each point is the mean ± S.E.M. (% of arbitrary fluorescent units) of 3–4 animals and each experiment (*n* = 3) was carried out at least in triplicate. The statistical analysis used was the Student *t* test for each antibody; ** *p* < 0.01 vs. CNT.

Glial fibrillary acidic protein (GFAP) is a widely known marker of activated mature astrocytes, whereas ionised calcium-binding adaptor molecule 1 (Iba-1) is used to study the activation of microglia. In the present study, hippocampal levels of GFAP and Iba1 were determined by immunohistochemistry. After AVCRI104P3 treatment astrogliosis was not modified, whereas the hippocampal levels of Iba-1, the marker of microgliosis, significantly decreased (34%; *p* < 0.05) compared to the control group (Figure 6).

We also analysed the influence of AVCRI104P3 on the expression of DCX, a protein that is found in the cytoskeleton of immature neurons and is used as a neurogenesis marker. In the present experimental conditions, no differences were observed between control and treated groups (Figure 6).

### 2.3. Effect of AVCRI104P3 on AChE isoforms (AChE-R, AChE-S)

**Figure 7:**
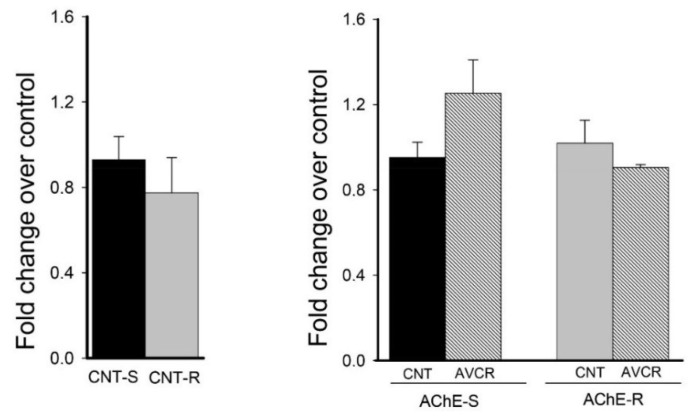
Effects of AVCRI104P3 on mRNA AChE-S and AChE-R expression in the pre-frontal cortex. (left) mRNA control levels (mRNA-S expression was considered 100%), (right) mRNA-S and mRNA-R expression in the pre-frontal cortex after AVCRI104P3 treatment. The mRNA expression levels were normalised to those of β-actin and considered to be 100% for control mice. Each point is the mean ± S.E.M. of 3–5 animals, and each experiment was carried out in triplicate. The statistical analysis used was one-way ANOVA followed by Dunnett’s test.

It is known that AChE expression decreases with aging and a correlation exists between AChE content and cognition [25]. Changes in the expression of AChE splice variants have been associated with responses to different stimuli and also with aging [26]. Before studying the modulation of the mRNA expression of the S and R variants caused by the administration of the drug, we evaluated the basal levels of both AChE-S (neurotoxic role) and AChE-R (neuroprotective role) variants. We did not find significant differences between the basal levels of both variants, with mRNA-S levels being only 16% higher than those of mRNA-R (Figure 7). Likewise, when evaluating the effect of the drug on the levels of the mRNA of the AChE variants, there were no significant differences between control and treated group, despite the upward trend in mRNA-S levels after AVCRI104P3 administration.

## 3. Discussion

Previous in vitro studies supported the idea that the multitarget hybrid compound AVCRI104P3 could behave as a disease-modifying agent, as it concurrently modulates different proteins involved in neurodegenerative processes [19]. More recently we have demonstrated that AVCRI104P3 induced cognitive improvement as well as anxiolytic effects [21]. It is known that aged brain is more prone to events associated with neuroinflammation, apoptosis and other damage processes [27], which mostly affect areas such as hippocampus and cortex [28]. In this context, herein we have studied the effects of AVCRI104P3 on proteins involved in survival and cellular death processes, as well as plasticity, synaptic function, gliosis and neurogenesis in both hippocampus and cortex of 12-month-old 129/Sv × C57BL/6 male mice.

The expression of the anti-apoptotic protein Bcl2 was significantly increased in both hippocampus (113%, *p* < 0.01) and cortex (39%, *p* < 0.01) after AVCRI104P3 treatment. Likewise, the levels of the phosphorylated form of Akt1 were increased (75%, *p* < 0.01), but only in the hippocampus. It has been demonstrated that the parent compounds from which the hybrid compound AVCRI104P3 was designed, namely huprines and donepezil, display neuroprotective effects mostly related to their ability to interact directly or indirectly with nicotinic receptors [29,30]. It is known that the α-7 nicotinic receptor subtype activates the PI3K/Akt pathway, which is involved in the regulation of the Bcl2 family, with the increase of Bcl-2 expression being one of the main anti-apoptotic effects [31]. Furthermore, we also studied the effects of AVCRI104P3 on the activation of the Akt/GSK3β pathway, where phosphorylation of GSK3β serine 9 by Akt is the main mechanism of inactivation of this protein. GSK3β is involved in the phosphorylation of tau among other proapoptotic processes. In the present study, we have observed an increase in the levels of the phosphorylated or inactive form of GSK3β (37%) in the hippocampus, to the detriment of the non-phosphorylated or active form thereof. These results support the involvement of the PI3K/Akt pathway-through the stimulation of α7 nicotinic receptors as a likely neuroprotective mechanism associated with AVCRI104P3.

Synaptophysin is an integral membrane protein of synaptic vesicles that is involved in the mechanisms of neurotransmitter release and synaptic plasticity [32]. After AVCRI104P3 administration we observed a slight but significant decrease of synaptophysin expression in both hippocampus (11%, *p* < 0.01) and cortex (7%, *p* < 0.05). However, a significant increase in synaptophysin expression was previously reported after huprine [13] and donepezil [33] treatment. Differences among the pharmacological profiles of the multitarget hybrid compound AVCRI104P3 and the parent compounds huprine and donepezil or among the animal models used in the different studies might account for these conflicting results.

CDK5 activation is involved in neuronal plasticity processes. It is known that inactivation of this protein plays an important role in the pathogenesis of neurodegenerative diseases. In the present study, we have found that the p25/p35 ratio, which is representative of the inactivation/activation ratio of CDK5, was not significantly modified after chronic treatment with AVCRI104P3. Similar results were reported for huprines and the structurally related drugs tacrine and huperzine A, which did not modify the CDK5 deregulation induced by colchicine in cerebellar granule neurons [34].

The activity of caspases 3 and 7, other important proteins in the cascade of apoptotic events, was not modified with the administration of the anticholinesterasic AVCRI104P3. Contradictory results have been reported in a number of studies about the relationship between AChE inhibitors and caspases. Thus, it has been reported that donepezil induced caspase activity in leukemia HL-60 cells [35], whereas other reports described an inhibition of caspases by the AChE inhibitors huperzine A [36], galantamine [37], and donepezil [38], which led to neuroprotective effect in different experimental models. Controversial results have also been reported about the relationship between caspase activity and aging process. Thus, Means et al. [39] observed that normal aged mice (24 months) showed behavioral cognitive impairment with a concomitant high caspase activity compared to young animals (6 months) of the same strain, whereas other authors did not find any change in brain caspase activity in mice of 6 to 24 months of age [40]. In this light, it could be argued that in our 12-month mouse model the activity of caspase is not altered enough as to allow us to observe any effect of the drug.

Brain aging is generally associated with a certain level of neuroinflammation [41,42] and a decrease in neurogenesis [43]. Under these conditions, there is an increase in brain inflammatory mediators that induce the activation of microglia and astrocytes [42] and further neurogenesis declines [43]. Different studies have reported that some AChEIs can modify the inflammatory response in different conditions [44,45]. In this study, it was determined whether the anticholinesterasic AVCRI104P3 could modify the expression of cerebral inflammatory protein markers such as GFAP and Iba1 as it is known that activation of nicotinic receptor is involved in anti-inflammatory mechanisms [46]. Indeed, we previously observed an anti-inflammatory effect of huprine X in the kainic acid mouse model [14]. In this work, we have observed that AVCRI104P3 did not modify the astrogliosis marker (GFAP), even though it significantly reduced the expression of the activated microglia protein marker (Iba-1) (34%). This anti-inflammatory effect of AVCRI104P3 could be explained through two mechanisms, i.e., by AChE inhibition and/or by activation of α7 nicotinic receptors involved in the anti-inflammatory cholinergic pathway [46], with the subsequent inhibition of TNFα, among other proinflammatory cytokines [47,48]. The involvement of muscarinic receptors cannot be ruled out, as it has been observed that stimulation of M1 receptors inhibits the production of TNFα and activates efferent vague nerve, and, consequently, the release of ACh is increased and α7 nicotinic receptors are activated [49].

Neurogenesis decreases during neurodegenerative disease and the aging process as well. As it was observed in the case of huprine X [14], treatment with AVCRI104P3 did not change the expression of the neurogenesis marker DCX in the DG of the hippocampus under the present experimental conditions. This is in contrast to previous reports where it was observed that the AChEIs donepezil, rivastigmine, and galantamine were able to stimulate neurogenesis in different mouse models [50]. The lack of response of DCX to the anticholinesterasic AVCRI104P3 could indicate that other factors than AChE inhibition might be involved in the neurogenesis induced by donepezil, rivastigmine, and galantamine.

Finally, the effect of AVCRI104P3 on AChE mRNA levels of splice isoforms S and R was evaluated, as they are associated to both pro- or anti-apoptotic processes, respectively. When we compared the basal mRNA levels of both variants, we observed that levels of AChE-S were not significantly different than those of AChE-R. However, in adult mice the S-isoform seems to be the most abundant form of AChE. It has been described that AChE activity decreases between 30 and 50% in 18-month-old male rat brains compared to those of 3 months and that the S-isoform is more vulnerable to aging than the R-isoform [26]. In addition, in demented patients the AChE-R isoform seems to increase significantly with regard to control group [51]. When evaluating the effect of the drug on both AChE isoforms no significant changes in mRNA-S and mRNA-R levels were observed in our experimental approach. In fact, it has been pointed out that a specific pattern of response to AChEIs does not exist, given that donepezil is associated with a decrease of the AChE-R/AChE-S ratio while rivastigmine seems to increase it [52]. 

In summary, in the present study we have demonstrated that AVCRI104P3 activates the expression of several proteins involved in the anti-apoptotic process such as Akt, Bcl2 and pGSK3β and furthermore, it shows an important anti-inflammatory effect through inhibition of microglia. Overall, these results suggest that the positive behavioral effects previously reported after AVCRI104P3 treatment [21] could be partly related to the neuroprotective mechanisms unveiled in this study, which further support the potential of this compound for the treatment of brain dysfunction associated with aging and/or dementia.

## 4. Materials and Methods

### 4.1. Subjects and Drug Treatment

**Figure 8:**
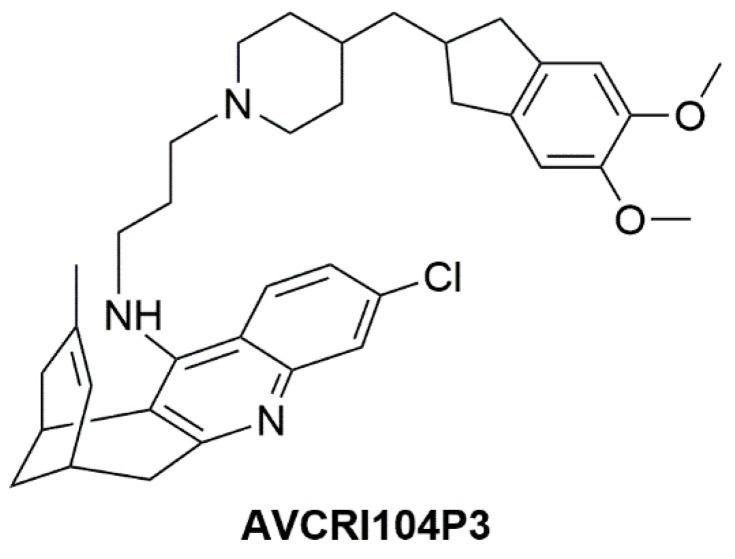
Chemical structure of AVCRI104P3.

In the present study, eighteen 12-month-old 129/Sv × C57BL/6 male mice were used. The animals were maintained in the facilities of the Medical Psychology Unit, Facultat de Medicina, Universitat Autònoma de Barcelona. Three animals were housed per cage in standard plastic type Macrolon cages (35 cm × 35 cm × 19 cm, with 2 L of wood cuttings as bedding) up to the time of the experiment and maintained under standard conditions: temperature 22 ± 2 °C; relative humidity 60 ± 10%; 12 h light/dark (lights on at 08:00 h); lab chow and tap water ad libitum. AVCRI104P3, (±)-3-chloro-12-[(3-{4-[(5,6-dimethoxyindan-2-yl)methyl]piperidin-1-yl}propyl)amino]-6,7,10,11-tetrahydro-9-methyl-7,11-methanocycloocta[*b*]quinoline [19] (Figure 8) was synthesized at the Laboratory of Pharmaceutical Chemistry, Faculty of Pharmacy and Food Sciences, University of Barcelona. 

Animals were distributed in two groups (*n* = 8/group) and received chronic treatment with 0.9% of saline solution (control group) or AVCRI104P3 (0.6 µmol/kg/day; 0.43 mg/kg/day; treated group) intraperitoneally. The drug was dissolved in a vehicle of 0.9% saline solution. Mice were sacrificed by decapitation after 21 days of treatment and brains were quickly removed and hemisected on ice (4 °C). Finally, hippocampus and cortex were dissected for Western blot analysis and 5 half-brains (each group) were fixed in paraformaldehyde for immunohistochemistry studies. Thereafter, all samples were frozen at −80 °C until subsequent analysis.

All animals were treated according to protocols approved by Department of the Environment and Housing (DMAH, Generalitat de Catalunya, SPAIN) in 16 March 2014 (certificate No: DMAH-7981). All the research was conducted in compliance with the Spanish legislation on “Protection of Animals Used for Experimental and Other Scientific Purposes” and in accordance with the EU Directive 2010/63/EU on this subject. Besides, the study complies with the ARRIVE guidelines developed by the NC 3Rs and the efforts to reduce the number of subjects used [53].

### 4.2. Western Blotting (WB)

Hippocampus or cortex were individually homogenized (Polytron; 4 °C) in a buffer solution: 1 M Tris-HCI (pH 7.5), 0.5 M EDTA (pH 8); 1% Triton X-100, protease inhibitor cocktail and phosphatase inhibitor cocktail, using a 1:20 dilution. Then, samples were centrifuged at 12,000× *g* for 10 min at 4 °C and the supernatant was frozen at −80 °C.

The procedure for the quantification of protein expression for immunoblotting was done as previously described [14]. In this study the following primary antibodies were used: Akt1 (1:1000; Sigma, St. Louis, MO, USA); PAkt1 (Ser473) (1:1000; Sigma); Bcl2 (1:1000; Bd Biosciences, New York, NY, USA); GSK3β (1:1000; Cell Signaling, Boston, MA, USA); P-GSK3β (Ser9) (1:1000; Cell Signaling); p35 (C-19) (1:1000; Santa Cruz, Santa Cruz, CA, USA); p25 (1:1000; Santa Cruz); synaptophysin (1:2000; Dako, Glostrup, Denmark); β-actin (1:10,000; Sigma). Horseradish peroxidase was used as a secondary antibody and β-actin was assayed simultaneously using the β-actin monoclonal antibody as the primary antibody.

The immunoblots were developed with WB detection ECL reagents. Quantification of results was carried out using the GeneSnap program and the optical density of each sample was corrected using the optical density of the corresponding β-actin band.

Samples from treated mice were evaluated as a percentage of the control group (saline) for each experiment (WB membrane). Values are expressed as the mean of the percentages compared to the control group.

### 4.3. Immunohistochemistry

Immunohistochemistry was performed by the free-floating method. For this purpose, half-brains (*n* = 5) were fixed in 4% paraformaldehyde in phosphate buffered saline (PBS) pH 7.4; 0.137 M NaCl; 0.0027 M KCl; 0.02 M Na_2_HPO_4_·2H_2_O; 0.1 M K_2_HPO_4_) and maintained at 4 °C for 24 h. Thereafter, brains were rinsed 3 times in 30% sucrose solution in PBS and frozen by isopentane. Sagittal sections of 20 µm were maintained in freeze-solution (PBS 30%; ethylene glycol 40%; glycerol 30%) at −20 °C until the ulterior analysis. The following antibodies were used in the immunohistochemistry studies: GFAP (1:3000; Dako); Doublecortin (1:2000; Santa Cruz); Iba-1 (1:200; Abcam, Cambridge, UK); Cyn3 (1:400; rabbit; Jackson, West Grove, PA, USA); Alexafluor 488 (1:400; rabbit; Jackson).

Sagittal sections were obtained as previously described [14]. Brain sections were mounted on slides (6 cuts per slide). Labelling was observed under fluorescent light at optimal sensitivity using an appropriate filter system. The 20× images were analysed by Image J 1.49 free Software. Samples without primary antibody were used as non-specific binding and used as the background for the evaluation of the three experimental groups. Samples from treated mice (AVCRI104P3) were evaluated as a percentage of arbitrary fluorescence units of the control group.

### 4.4. Caspase Activity

Caspase-3/7, caspase-8 and caspase-9 activities were measured using the corresponding Caspase-Glo Assay kits (Promega, Madison, WI, USA) as previously described [14]. Luminescence units were used to evaluate the effects of drug treatment on caspase activity.

### 4.5. RT-PCR

According to Livneh et al. [54], AChE-R transcripts highest induction by IAChE takes place in mice frontal cortex. Therefore, we used frontal cortex for AChE-R and AChE-S mRNA analysis.

Total RNA was extracted and isolated using PureLink^TM^RNA Mini Kit (Life Technologies, Carlsbad, CA, USA) following the manufacturer’s protocol. Treatment with RNase-free DNase I (Life Technologies) removed traces of DNA from the samples.

Pure RNA (10 µg) was retrotranscribed and amplified using AChE-R and AChE-S primers [55] (Invitrogen, Life Technologies) along with SYBR^®^ Green PCR Mix (Life Technologies) in a StepOne^TM^ Real-Time PCR Biorad CFX96 System according to the manufacturer’s instructions (5 min, 50 °C; 40 cycles, 15 s, 95 °C; 1 min, 60 °C).

Transcript levels for AChE (-R, -S) were calculated using the relative standard curve method normalized to β-actin [56]. Control mice mRNA levels were considered 100%.

### 4.6. Statistics

Results were expressed as means ± SEM using 4–5 mice per group of treatment. The Student *t*-test for unpaired samples and one-way ANOVA followed by Dunnett’s test were used, according to the experimental approach. Statistical significance was considered at *p* < 0.05 (GraphPad Prism 4).
